# Response to: Best practice advice on pre-hospital emergency anaesthesia & advanced airway management

**DOI:** 10.1186/s13049-019-0613-7

**Published:** 2019-03-18

**Authors:** Jostein S. Hagemo, Per P. Bredmose, Halvard Stave, Marius Rehn, Christian Buskop

**Affiliations:** 1 Division of Prehospital Services, Air Ambulance Department, Oslo University Hospital, Oslo, Norway; 2 Department of Research. Norwegian Air Ambulance Foundation, Oslo, Norway; 3 Faculty of Health Sciences, University of Stavanger, Stavanger, Norway

**Keywords:** Pre-hospital emergency anaesthesia, Airway management, HEMS, Competence

## Abstract

The European HEMS and Air ambulance Committee’s Medical working group recently published Best Practice advice on pre-hospital emergency anaesthesia and advanced airway management. We believe that this initiative is important. In our opinion however, the competence requirements recommended by the authors do not meet the standards that we should aim for in HEMS services. We argue that pre-hospital emergency anaesthesia should be delivered with a competence level approximating in-hospital standard. In our experience, our patients benefit from pre-hospital emergency anaesthesia delivered by consultants with regular in-hospital rotations and a sound clinical governance system.

Dear Sir,

We would like to express our gratitude for the Best Practice Advice (BPA) initiative of the European HEMS and Air ambulance Committee’s Medical Working Group (EHAC MWG). We take great interest in their most recent BPA on pre-hospital emergency anaesthesia (PHEA) & advanced airway management [1]. Setting standard requirements for these interventions is an important step towards assuring high-quality delivery of pre-hospital critical care. We acknowledge the major challenges EHAC MWG faces in formulating BPAs aiming to encompass systems with major differences in operative environment, organization and tradition. Nevertheless, we would like to comment on the competence requirement issues discussed in the BPA.

PHEA is typically performed in the sickest of patients, where clinical information is limited, and the environment is austere. In our opinion, these are exactly the situational characteristics that call for senior skills, experience and operational awareness.

The current BPA recommends a minimum of 80 supervised endotracheal intubations before attempting PHEA. Apparently, the evidence supporting this recommendation is limited to one study that enrolled eleven junior physicians and, importantly, excluded patients with an anticipated difficult airway [2]. Still, after 80 intubations, two out of eleven subjects were unable perform intubation without the physical assistance from a consultant. We question whether the existing evidence allows for recommending a minimum standard of only 80 intubations before attempting PHEA. In contrast, studies from HEMS in our region document successful pre-hospital intubation in 99% of cases when performed by an experienced anaesthesiologist [3]. In most pre-hospital scenarios, physical senior assistance is not readily available and PHEA should accordingly be left to providers with senior skills and experience.

More importantly is our concern that the issue of competence among professionals delivering PHEA is reduced to a debate on correctly placing an endotracheal tube. This would be a failure to acknowledge the complexity of an intervention with potential serious side effects in critically ill patients. Studies report high incidence of hypotension and hypoxemia following PHEA [4, 5]. These are complications that unquestionably have negative long-term effects in certain groups, but may still only be the tip of the iceberg. A number of physiological side effects of PHEA are less obvious, but still have potentially major impact on outcome [6].

Based on experience from HEMS in the south-eastern part of Norway, our patients benefit from receiving PHEA governed to a standard of care equivalent to in-hospital best practice. This implies utilizing advanced diagnostic tools, invasive monitoring and vasoactive infusions whenever appropriate. Induction and maintenance of anaesthesia is tailored to the patient’s physiological condition, including any recognised co-morbidities. We believe this can only be achieved by highly experienced providers maintaining their skills through regular in-hospital service rotations supported by a sound clinical governance system.

In order to meet the challenges in the wide range of critical care conditions encountered in pre-hospital care, the Air Ambulance Department at Oslo University Hospital require that pre-hospital physicians have completed in-hospital rotations in cardiothoracic-, neurosurgical-, and paediatric anaesthesia as well as intensive care medicine as part of the consultant qualification program. Due to a relatively large proportion of emergency neonatal transport missions in our service, we also require significant experience from neonatal intensive care. Senior consultants with expertise in pre-hospital ECMO and iNO transportations are available for advice at all times. With the current case mix, the disease severity and the transport distances in our catchment area, we question whether one year of anaesthesia practice and one year of emergency medicine practice, as suggested by the BPA authors, would be sufficient in delivering PHEA with a quality approximating in-hospital standards.

When giving advice on best practice in HEMS service, one should aim for senior care and avoid solutions based on minimal requirements. Our HEMS service has developed from applying junior doctors to a mature consultant led system over decades. We firmly believe this transition has been to the benefit of our patients.

## Abbreviations

BPA: Best practice advice
ECMO: Extra corporal membrane oxygenation
EHAC: European HEMS and Air ambulance Committee
HEMS: Helicopter Emergency Medical Service
iNO: inhaled Nitric Oxide
MWG: Medical Working Group
PHEA: Pre-hospital Emergency anaesthesia
